# Late presentation to HIV/AIDS care in Brazil among men who self-identify as heterosexual

**DOI:** 10.1590/S1518-8787.2016050006352

**Published:** 2016-08-16

**Authors:** Sarah MacCarthy, Sandra Brignol, Manasa Reddy, Amy Nunn, Inês Dourado

**Affiliations:** I RAND Corporation. California, USA; II Universidade Federal Fluminense. Niterói, RJ, Brasil; III The Miriam Hospital. Alpert Medical School of Brown University. Rhode Island, USA; IV School of Public Health of Brown University. Rhode Island, USA; V Instituto de Saúde Coletiva. Universidade Federal da Bahia. Salvador, BA, Brasil

**Keywords:** Men’s Health, Heterosexual, Acquired Immunodeficiency Syndrome, Cross-Sectional Studies, Late Presentation, HIV/AIDS

## Abstract

**OBJECTIVE:**

To analyze the factors associated with late presentation to HIV/AIDS services among heterosexual men.

**METHODS:**

Men infected by HIV who self-identified as heterosexual (n = 543) were included in the study. Descriptive, biivariate and logistic regression analyses were performed to evaluate the factors associated with late presentation (defined as individuals whose first CD4 count was <350 cells/mm^3^) in the study population.

**RESULTS:**

The prevalence of late presentation was 69.8%. The multivariate logistic analysis showed testing initiated by the provider (OR_adjusted_ 3.75; 95%CI 2.45–5.63) increased the odds of late presentation. History of drug use (OR_adjusted_ 0.59; 95%CI 0.38–0.91), history of having sexually transmitted infections (OR_adjusted_ 0.64; 95%CI 0.42–0.97), and having less education (OR_adjusted_ 0.63; 95%CI 0.41–0.97) were associated with a decreased odds of LP.

**CONCLUSIONS:**

Provider initiated testing was the only variable to increase the odds of late presentation. Since the patients in this sample all self-identified as heterosexual, it appears that providers are not requesting they be tested for HIV until the patients are already presenting symptoms of AIDS. The high prevalence of late presentation provides additional evidence to shift towards routine testing and linkage to care, rather than risk-based strategies that may not effectively or efficiently engage individuals infected with HIV.

## INTRODUCTION

Globally, studies have documented unprotected receptive anal intercourse (URAI) as an important risk factor for the transmission of HIV^3^. Consequently, men who self-identify as heterosexual are often overlooked in efforts to address HIV/AIDS, as it is assumed that they are not engaging in URAI. However, recent literature has documented high numbers of heterosexual men presenting to care with low CD4 cell counts^12^ ^,^ ^17^. Commonly referred to as late presentation (LP) to care^2^, data from high-income countries show the prevalence of LP to range between 52.5% and 67.2%^8,15,20-23.^. Less is known about the prevalence in middle or low-income countries, but studies from Brazil – one of the first countries to guarantee free, universal antiretroviral (ARV) since 1996 in the Brazilian Unified Health System (SUS) and an expected associated opportunity to improve access to care – have shown the prevalence ranging from 43.6% to 54.0%^10^ ^,^ ^18^.

Studies noted an association between men who self-identify as heterosexual and LP globally^3^ ^,^ ^4^ ^,^ ^6^ ^,^ ^8^ ^,^ ^19^ and in Brazil^10^, but few of these studies identified the factors specifically associated with LP among this population. In the general population, LP is more frequent among males^4^ ^,^ ^8^ ^,^ ^23^, older adults^4^ ^,^ ^5^ ^,^ ^11^ ^-^ ^15^ ^,^ ^25^, and immigrants^5^ ^,^ ^11^ ^,^ ^13^ ^,^ ^15^ ^,^ ^23^ ^,^ ^25^. Fewer studies have assessed predictors of LP by sexual identity, more often focusing on sexual behavior. However, recognizing that same-sex behavior is often stigmatized, and that patients may not be willing to disclose behavior to their medical providers, identifying factors associated with LP by self-reported sexual identity could be useful to clinicians who must rely on patient report.

Thus, the research question of this study was to identify the prevalence and predictors of LP among self-identified heterosexual men (hereafter referred to as heterosexual men) living with HIV.

We explored LP among heterosexual men in Brazil, an upper middle-income country often considered a global model of success for their response to HIV with one of the largest antiretroviral treatment programs in the world, to expand the knowledge regarding this important public health issue in the care cascade for HIV/AIDS.

## METHODS

In this cross-sectional study, we collected data on 1,970 participants (men = 1,056 and women = 914) in Northeast Brazil. All participants were HIV-infected (confirmed by laboratory diagnosis), aged 18 years or older, and enrolled for clinical care for the first time at one of three main health facilities in Salvador, BA, Northeastern Brazil, from August 2010 to June 2011. Responding to growing evidence in the peer-reviewed literature that men who identify as heterosexual present increased risk for LP, we limited our analysis to all men who self-identified as heterosexual (n = 591) to the question on sexual identity.

The facilities included the public HIV/AIDS specialty care center and two large public hospitals providing general and HIV/AIDS outpatient care. Since 1997, the Brazilian government has provided HIV/AIDS care and treatment free of charge at all facilities belonging to the SUS. The facilities were located in Salvador, a large urban center of Brazil, capital of Bahia, and the third most populous (approximately 2.7 million people) and poorest city in the country.

The study staff attended the HIV specialty care center and the HIV outpatient care at the two hospitals daily. A list of scheduled patients was provided to the study team beforehand. Refusal to participate was minimal (less than 5.0%). All patients were counseled that the participation in the study entailed responding to our survey and allowing access to their laboratory data. Further, the participants were asked to sign an informed consent form. The interviews were individually conducted in a private space at the facility with trained research staff. Data were collected by oral interviews. Patients were asked a range of questions, and responses were recorded using a palm pilot. The interview addressed comprehensive questions on sociodemographic characteristics, access to HIV/AIDS services, as well as sexual and other behaviors associated with LP. No financial incentives were provided.

The outcome variable, LP, was based on the consensus statement released by the European Late Presenter Group, which defined LP as individuals with a CD4 cell count < 350 cells per mm^3^ or symptoms of AIDS-defining illnesses, or both^2^. Here, LP was restricted to individuals with available CD4 cell count because we lacked data to identify individuals defined as an AIDS case based on clinical symptoms. As a result, 9.2% men were excluded, reducing the sample size to n = 543. A subanalysis confirmed that individuals with no clinical data were similar to the sample population. To complete and validate data on the first CD4 cell count, trained research assistants used information from hand written clinical records and from the *Sistema de Controle de Exames Laboratoriais da Rede Nacional de Contagem de Linfócitos CD4+/CD8+ e Carga Viral* (SISCEL – Laboratory Test Control System of the Brazilian Network of CD4+/CD8+ T Lymphocyte and Viral Load Count) national database. This system is organized by patient identifier code and the database is powered by a nationwide network of reference laboratories^10^.

Key variables to the study of LP were explored. Age was dichotomized based on the traditional cut point for reproductive age (18-44 years *versus* 45-86 years). Skin color was categorized into black, brown, and white, yellow, indigenous, and other. The remaining sociodemographic variables were dichotomized as follows: individual income (comparing individuals receiving minimum wage of BRL510.00 per month = USD328.11 per month or less to individuals receiving above it); employment (comparing individuals formally and informally employed to individuals who were unemployed); and years of schooling (comparing individuals receiving the minimum eight years of schooling required by the Brazilian government to individuals receiving more). Regarding other factors associated with LP in the peer-reviewed literature, we examined reasons for taking an HIV test (comparing individuals requesting an HIV test to individuals who were requested by their provider to test for HIV); perceived risk for HIV transmission (yes; no); history of drug use (comparing individuals who already reported drug use to individuals who never reported drug use); number of sexual partners in the last 12 months (comparing individuals reporting one or less to individuals reporting more); and history of sexually transmitted infection (STI) (comparing individuals who never had a diagnosed STI to individuals who had a diagnosed STI at least once).

Descriptive and bivariate analyses were conducted and statistically significant variables (determined by the 95% confidence interval – CI of the odds ratio – OR) were included in the final multiple logistic regression analysis along with key variables highlighted in the literature. We used logistic regression analyses with adjusted odds ratios (AOR) to evaluate the independent effect of potential explanatory variables and model diagnostics tests to examine associations and outliers. The analyses used the Stata software, version 12. The goodness of fit of the model was verified by the Hosmer-Lemeshow chi-square test, comparing the expected frequencies with the observed frequency (p = 0.35), which indicated that the model fits the data well. The area under the ROC curve of 0.71 showed acceptable discrimination between LP and not LP. The model also showed good agreement (72.50) and high sensitivity (92.1).

The Research Ethics Committee of the Bahia State Secretariat of Health and of Harvard School of Public Health approved this study.

## RESULTS

The prevalence of LP based on the consensus definition was 69.8% among heterosexual men. The prevalence of individuals with CD4 below 200 was 50.1%. Of note, 86.1% presented a CD4 count ≤ 500 cells/mm^3^, the new threshold for treatment initiation in Brazil since 2013. In terms of the distribution of initial CD4 counts, the Figure shows that the average CD4 count was 322 cells/mm^3^ for men in reproductive age, compared to 244 cells/mm^3^ among men aged 45 and older.

**Figure:**
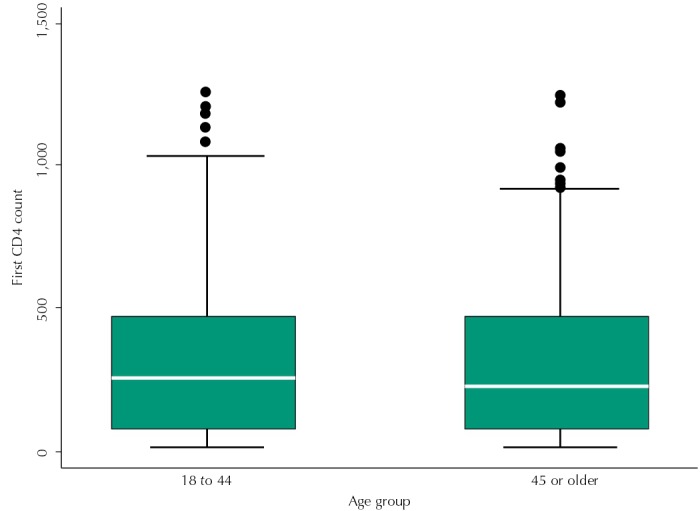
Distribution and average CD4 count for men who self-identify as heterosexual, by age groups 18 to 44 (322 cells per mm3) and 45 or older (242 cells per mm3).

Full descriptive statistics are reported in Table 1. In our sample, 47.0% self-identified as brown, 33.0% as black, and 21.0% as white, yellow, indigenous, or other. Just over half of respondents were 44 years old or younger. Half of respondents were employed, and 76.0% reported an income above the official minimum wage. Nearly half of respondents had completed more than eight years of education. Further, 69.0% and 60.0% reported no history of drug use and STI, respectively. Approximately 36.0% of all respondents reported having more than one sexual partner in the past 12 months.

**Table 1:** Descriptive data of men self-identified as heterosexual receiving HIV/AIDS care in Brazil.

Variable	Men who self-identify as heterosexual	
	(n = 543)	

	n	%
CD4 cell count at presentation to care		
< 350 cells/mm^3^	379	69.8
≥ 350 cells/mm^3^	164	30.2
Reported sexual practice		
Reported sex with women only	285	52.5
Reported sex with women and men	258	47.5
Age (years)		
18-44	309	56.9
45-86	234	43.1
Skin color		
Brown	253	46.7
Black	177	32.7
White, Yellow, Indigenous, or other	112	20.6
Individual income*		
> Minimum wage	415	76.4
≤ Minimum wage	118	23.6
Employment		
Employed	270	49.8
Unemployed	272	50.2
Years of schooling		
> 8 years	237	43.7
≤ 8 years	306	56.3
Reason for taking an HIV test		
Patient requested the HIV test/Other	203	37.4
Provider requested the HIV test	340	62.6
Perceived HIV risk		
No	411	76.0
Yes	130	24.0
History of drug use		
No	373	68.7
Yes	170	31.3
Number of sexual partners in the last 12 months		
≤ One	329	63.8
> One	187	36.2
History of STI		
Never	322	59.9
At least once	216	40.1

STI: sexually transmitted infections

* Minimum wage of BRL510.00 per month (USD328.11 in 2011), as established by the Brazilian government.

The bivariate results highlighted several variables associated with LP (Table 2). Individuals who were unemployed (OR = 1.60; 95%CI 1.10–2.32), whose provider requested they be tested for HIV (OR = 4.02; 95%CI 2.68–6.05), and who perceived they were at risk for HIV (OR = 1.52; 95%CI 1.04–2.22) were all associated with increased odds for LP. In contrast, individuals reporting less education (OR = 0.79; 95%CI 0.41–0.89) and history of drug use (OR = 0.61; 95%CI 0.41–0.89) were associated with a decreased risk for LP.

**Table 2:** Number, proportion and odds ratio of late presentation among men self-identified as heterosexual receiving HIV/AIDS care in Brazil.

Variable	Late presentation among men who self-identify as heterosexual			

	n	%	OR	95%CI
Reported sexual practice				
Reported sex with women only	200	70.2	1.00	
Reported sex with women and men	179	69.4	0.96	0.67–1.39
Age (years)				
18-44	214	69.3	1.00	
45-86	165	56.6	1.06	0.73–1.54
Skin color				
Brown	171	67.6	1.00	
Black	126	71.2	1.18	0.78–1.80
White, Yellow, Indigenous, or other	81	72.3	1.25	0.77–2.05
Individual Income*				
> Minimum wage	290	70.0	1.00	
≤ Minimum wage	89	69.5	0.98	0.64–1.51
Employment				
Employed	175	64.8	1.00	
Unemployed	203	74.6	1.60	1.10–2.32
Years of schooling				
> 8 years	172	72.6	1.00	
≤ 8 years	207	67.7	0.79	0.41–0.89
Reason for taking an HIV test				
Patient requested the HIV test/Other	104	51.2	1.00	
Provider requested the HIV test	275	80.9	4.02	2.68–6.05
Perceived HIV risk				
No	123	64.1	1.00	
Yes	254	73.0	1.52	1.04–2.22
History of drug use				
No	273	73.2	1.00	
Yes	106	62.4	0.61	0.41–0.89
Number sexual partners in the last 12 months				
≤ One	234	71.1	1.00	
> One	126	67.4	0.84	0.57–1.24
History of STI				
Never	235	73.0	1.00	
At least once	140	64.8	0.68	0.57–1.23

STI: sexually transmitted infections

* Minimum wage of BRL510.00 per month (USD328.11 in 2011), as established by the Brazilian government.

Table 3 shows the multiple logistic regression results. Once controlling for additional variables of interest, provider-initiated testing was the only variable to increase the odds for LP (OR = 3.82; 95%CI 2.49–5.85). Having a history of drug use (OR = 0.58, 95%CI 0.37–0.91) or past STI (OR = 0.62, 95%CI 0.40–0.95), and reporting less education (OR = 0.63, 95%CI 0.41–0.97) were associated with decreased odds for LP.

**Table 3:** Predictors of late presentation: adjusted odds ratios and 95%CI of late presentation among men self-identified as heterosexual receiving HIV/AIDS care in Brazil.

Variable	Late presentation among men who self-identify as heterosexual			

	n	%	OR	95%CI
Reported sexual practice				
Reported sex with women only	200	70.2	1.00	
Reported sex with women and men	179	69.4	1.51	0.85–2.69
Age (years)				
18-44	214	69.3	1.00	
45-86	165	70.5	0.93	0.60–1.42
Skin color				
Brown	171	67.6	1.00	
Black	126	71.2	1.58	0.98–2.53
White, Yellow, Indigenous, or other	81	72.3	1.23	0.71–2.13
Employment				
Employed	175	64.8	1.00	
Unemployed	203	74.6	1.52	0.99–2.31
Years of schooling				
> 8	172	72.6	1.00	
≤ 8	207	67.7	0.63	0.41–0.97
Reason for taking an HIV test				
Patient requested the HIV test/Other	104	51.2	1.00	
Provider requested the HIV test	275	80.9	3.82	2.49–5.85
Perceived HIV risk				
No	298	72.5	1.00	
Yes	80	61.5	0.87	0.54–1.41
History of drug use				
No	273	73.2	1.00	
Yes	106	62.4	0.58	0.37–0.91
Number sexual partners in the last 12 months				
≤ One	234	71.1	1.00	
> One	126	67.4	0.67	0.37–1.20
History of sexually transmitted infection				
Never	235	73.0	1.00	
At least once	140	64.8	0.62	0.40–0.95

## DISCUSSION

The analysis highlighted that provider-initiated testing was associated with increased odds for LP among heterosexual men, while experience with drug use, history of STI, and lower levels of education decreased the odds for LP.

The prevalence of LP among heterosexual men in our sample was similar to levels observed by Mocroft et al.^17^ in the COHERE study (66.1%) and by Iwuji et al.^12^ in the UK (68.8%), which appears higher than noted in the global literature in studies on the general population, found between 38.0% and 59.0%^1^ ^,^ ^5^ ^,^ ^7^ ^,^ ^8^ ^,^ ^19^ ^,^ ^24^ ^,^ ^25^. This could be due to the fact that our analysis is limited to men and several studies have noted that being male is associated with a higher risk for LP^18^, as women are more commonly tested during prenatal care and likely linked to care with a higher CD4 count compared to individuals testing for HIV only upon showing symptoms of opportunistic infections associated with HIV. The high prevalence of LP provides additional evidence to shift towards routine testing and linkage to care, rather than risk-based strategies that might not effectively or efficiently engage individuals infected with HIV.

The results highlighted that the provider-initiated testing, compared to patient-initiated testing, was associated with an increased odds ratio for LP. Given that Brazil has a concentrated epidemic, individuals are not referred for testing unless they present HIV or AIDS-related symptoms or report specific behaviors known to increase their risk of HIV transmission. Since all patients in this sample self-identified as heterosexual, it appears that providers are not requesting them to be tested for HIV until the patients are already presenting symptoms of AIDS. These results further suggest that testing should shift away from a risk-based strategy, as it likely ignores individuals who may not openly report certain behaviors traditionally associated with an increased risk of transmission.

Several variables were associated with decreased odds for LP. Experience with drug use has been shown in the global literature to be associated with an increased risk for LP^7^ ^-^ ^9^ ^,^ ^13^ ^,^ ^19^. However, one study that distinguished the stage in the HIV care to which individuals presented late found that drug users were more likely to be tested and less likely to present to care^9^. This suggests that more specific information, such as data related to late testing, enrollment, and treatment, may be needed to accurately identify to which service an individual is presenting late; also, individuals aware of their risk for HIV might be more likely to engage in HIV-related care, which was confirmed with our results, though the effect was not statistically significant in the multiple logistic regression.

Having been diagnosed with STI in the past was also associated with decreased odds for LP. This is likely related to the fact that individuals with a diagnosed STI have already come in contact with a service provider and therefore are more aware of their risk for HIV compared to other individuals^16^. This reflects how previous engagement with health services can impact LP. While professionals discuss how to organize and integrate HIV care with other health services, our results showed that the engagement with care, broadly defined, may improve the timing with which services are accessed.

We also observed that individuals with less education experienced slightly decreased odds for LP. This finding is counterintuitive, especially in light of our results that highlight how the awareness of risk for HIV can decrease the odds for LP. It is possible that the data reflects an underlying difference in the sample population. Since the study was conducted at a public clinic, it serves as the primary source of care for more marginalized individuals, whereas individuals who can afford private care do not seek services in the public sector until they need to access treatment. Without having access to data from privately funded facilities, we are unable to test this hypothesis. Consequently, the relationship between education and HIV risk is complex and warrants further exploration in subsequent research.

In sum, the prevalence of LP is alarmingly high among heterosexual men in our study. Though they are not traditionally viewed as a key population, these results suggest that they often fall off the radar of both policymakers and providers, and therefore represent a missed opportunity for timely presentation to care. Also, shifting from a risk-based to a routine strategy for HIV testing could help to engage people in care, with HIV testing as only the first, albeit crucial, step of linking into continued HIV care. More generally, the data showed that knowledge of potential risk for HIV may serve as an important driver for individuals to seek available services.

Concerning the study limitations, the outcome measure focuses exclusively on CD4 count, and therefore it remains unclear to which service the patient is presenting late. However, since it continues to be the consensus definition of LP, we agreed it provided a useful marker for evaluating LP to HIV services more generally. Further, given the cross-sectional nature of the study design, the results cannot be generalized to the Brazilian population nor can the causality be determined. Going forward more specific and nuanced data would help to better understand how the receipt of specific HIV services impacted the odds of presenting to care. At last, there is evidence to suggest that self-reported sexual orientation might not be consistent with the sexual practices of an individual. Nonetheless, it is critically important to report the risk factors associated with self-reported sexual orientation, as providers must be aware of the potential risks among their patients who identify as heterosexual males. Therefore, research must continue to investigate if the potential risks for LP may differ based on how patients present themselves to their provider.

Regarding its substantial strengths, to our knowledge, this is one of the only studies to exclusively focus on heterosexual men and show factors associated with LP in this population. This study provides important insight regarding factors associated with men who self-identify as heterosexual, a population continually overlooked in HIV prevention and treatment efforts. Further, this study has a large sample size and gives insight to the effectiveness of one of the world’s largest treatment programs for people living with HIV/AIDS. Finally, this is one of the few articles focusing on LP outside of a high-income context and suggests that the longstanding availability of prevention and treatment might not translate into the utilization of services.

In conclusion, the factors associated with LP among heterosexual men were the following: provider-initiated testing was associated with increased odds for LP while experience with drug use, history of STI, and lower levels of education decreased the odds for LP. These results highlight the importance of considering how a continued focus on the concept of key populations may miss a range of individuals in need of HIV testing and continued access to HIV services to achieve viral suppression. This study suggests that expanding the reach of HIV testing might help to identify and engage those who otherwise remain invisible.
